# Using prior knowledge from cellular pathways and molecular networks for diagnostic specimen classification

**DOI:** 10.1093/bib/bbv044

**Published:** 2015-07-02

**Authors:** Enrico Glaab

**Keywords:** biomarker modeling, pathway analysis, network analysis, machine learning, cross-study analysis

## Abstract

For many complex diseases, an earlier and more reliable diagnosis is considered a key prerequisite for developing more effective therapies to prevent or delay disease progression. Classical statistical learning approaches for specimen classification using omics data, however, often cannot provide diagnostic models with sufficient accuracy and robustness for heterogeneous diseases like cancers or neurodegenerative disorders. In recent years, new approaches for building multivariate biomarker models on omics data have been proposed, which exploit prior biological knowledge from molecular networks and cellular pathways to address these limitations. This survey provides an overview of these recent developments and compares pathway- and network-based specimen classification approaches in terms of their utility for improving model robustness, accuracy and biological interpretability. Different routes to translate omics-based multifactorial biomarker models into clinical diagnostic tests are discussed, and a previous study is presented as example.

## Introduction

In spite of the remarkable advances in biomedicine over recent decades, for a wide range of common, systemic and chronic diseases, precise molecular markers for early diagnosis are not yet available. In particular, for some of the most prevalent neurologic disorders (e.g. Alzheimer’s [1] and Parkinson’s disease [2]) and cancer types (e.g. colorectal cancer [3] and lung cancer [4]), diagnostic tools are often only applicable in the late symptomatic stages of the disease, provide insufficient sensitivity or specificity or fail to detect clinically relevant disease subtypes. As a consequence, inadequate therapies may be prescribed, treatment could often start too late to prevent irreparable damage and the development of new and more effective therapies for early intervention is hampered by misdiagnosed individuals in the study cohorts. While alternative diagnostic approaches cannot be expected to fully address all of these issues, improvements in only one of these aspects could already have significant benefits for patients. Even for infectious diseases with relatively unambiguous symptoms, new diagnostic tools may be required because during the incubation period a clear distinction between contagious and uninfected individuals is usually not possible (e.g. this has been a major issue during the recent Ebola virus disease outbreak in Africa [5]).

Classical molecular diagnostic tests measure the abundance of only a single biomolecule, e.g. to diagnose prostate cancer, plasma levels of the prostate-specific antigen (PSA) are commonly assessed. Although these approaches have advantages in terms of simplicity and costs, for complex and heterogeneous diseases, single-molecule biomarkers tend to provide insufficient accuracy and robustness across different patient cohorts (even the widely accepted PSA test for prostate cancer has a limited sensitivity of 72.1% according to a meta-analysis [6]). Building multivariate biomarker models derived from high-throughput omics measurements, e.g. using DNA or protein microarrays, would, in principle, offer the potential to account for diverse subtypes or facets of diseases, provided that the studied disorder is characterized by heterogeneous molecular manifestations. However, limitations of these high-throughput techniques (e.g. random noise and systematic biases, lack of robustness and reproducibility) and statistical limitations in the analysis of high-dimensional data (e.g. the multiple testing problem [7], feature redundancy and interdependence [8, 9] and issues summarized as the ‘curse of dimensionality’ [10, 11]) often prevent the discovery of genuine and reproducible patterns for diagnostic specimen classification.

In recent years, new bioinformatics approaches have therefore been proposed, which involve the integration of prior biological knowledge from cellular pathways and molecular networks into the model-building procedure. These approaches make use of prior observations, according to which, differences in pathway activity between diverse biological conditions tend to be more stable than differences in individual gene activity [12], and disease-associated biomolecular changes tend to be coordinated and display grouping patterns within molecular subnetworks [13]. The main rationale is that by exploiting these patterns as prior information when building prediction models, spurious discriminative structures in the data can be recognized and more robust and coherent patterns at the pathway- and network-level are selected instead of predictive features. Although a wide range of corresponding machine learning methods is already available, the benefits and limitations of different approaches are not always clear, and an overview on which strategies are adequate for which specific goals or applications is lacking.

Here, a review of recent network- and pathway-based statistical learning approaches for high-throughput omics data set analysis is provided, including a critical discussion of limitations and the added value of different methodologies for improving model robustness, accuracy and biological interpretability. Because not all diseases are characterized by aberrant molecular signatures, the application focus is on diagnostic approaches for diseases with complex molecular manifestations. First, methods using information from public cellular pathway definitions will be discussed, then approaches exploiting prior knowledge from genome-scale molecular networks. Next, common limitations of pathway- and network-guided predictive modeling are pointed out, as well as shortcomings related to the biospecimen origin, processing and storage and possible techniques to address or alleviate limitations in data analysis. Finally, the potential for translating omics-derived multivariate sample classification models into chip-based diagnostic devices is discussed, and a previous success story is presented.

## Pathway-based biomarker modeling

Cellular pathway definitions from manually curated databases, including KEGG [14], BioCarta [15], WikiPathways [16], Reactome [17] and PID [18], are frequently used knowledge sources for biological data interpretation. These pathways are typically designed in a subjective manner, often using incomplete information and inconsistent criteria for judging the relevance of putative pathway members (see ‘Limitations and possible solution strategies’ section), but this does not affect the methodological principles of pathway analysis, and results provided by robust analysis techniques are not significantly altered by small variations in pathway definitions. In particular, enrichment analysis methods [19–23] and pathway activity visualization tools [24, 25] are commonly applied to exploit information from these databases and obtain a better understanding of pathway-level molecular alterations in omics data. The utility of pathway information for biomarker discovery has also been recognized early [26], and more recently, a variety of bioinformatics approaches have been proposed to directly integrate knowledge from pathways into predictive model building for omics sample classification.

These methods mainly differ in terms of their pathway activity scoring approach (i.e. the approach used to summarize and score biomolecular activity data for cellular pathways as predictive features for classification tasks, e.g. via different dimension reduction methods, see discussion below) and the prediction method used (i.e. the machine learning algorithm applied to the summarized pathway activity scores to build a sample classification model). Table 1 shows an overview of different methodologies and their main characteristics, described in more detail as follows.

**Table 1.** Overview of pathway-based machine learning approaches for supervised classification of omics samples

Publication	Pathway activity scoring method	Prediction method
Guo *et al.* [27]	Mean or median expression levels in GO modules	Decision tree classification
Tomfohr *et al.* [28]	Expression levels are summarized via the first eigenvector from SVD analysis	Focus on predictive feature selection via *t*-statistics (any classifier is applicable)
Lee *et al.* [29]	Normalized sum of expression levels for CORGs within pathways	Logistic regression
Su *et al.* [30]	Weighted sum of LLRs for pathway members	Logistic regression or linear discriminant analysis
Glaab *et al.* [31]	Variance across pathway member activity is quantified and compared instead of averaged pathway activity	SVM, random forest, nearest shrunken centroid classifier or ensemble learning
Svenson *et al.* [32]	For each gene set and patient a ratio score is defined depending on the number of up- and down-regulated members in the gene set	Nearest centroids classification

Efroni *et al.* [33]	Joint scoring of pathway activity and consistency using interactions within pathways	Bayesian linear discriminant classifier
Vaske *et al.* [34]	Probabilistic inference is used to predict pathway activities from a factor graph model of genes and their products	Cox proportional hazard regression (survival prediction)
Breslin *et al.* [35]	Signal transduction pathways with directional interactions are used to define the activity of a pathway based on the average expression of its downstream target genes	No classification is performed, but contingency tables show associations between sample-wise pathway activity and clinical sample classifications
Kim *et al.* [12]	Hierarchical feature vectors are used, assigning a two-level hierarchical structure to the features/genes determined by their pathway membership	SVM

Averaging and dimension reduction approaches are listed on top, whereas graph-based and hierarchical pathway activity scoring methods are listed below the bold black line.

### Averaging and dimension reduction approaches to score pathway activity

One of the first machine learning approaches for high-throughput data analysis guided by pathway knowledge was proposed by Guo *et al.* [27]. Their method classified microarray cancer samples by computing mean or median expression levels of the gene members in biological process modules from the Gene Ontology (GO) database as input for a decision tree classifier [36]. This straightforward averaging approach was already reported to provide benefits in terms of robustness to measurement noise and comparable or better classification performance on cancer microarray data as compared with conventional gene-level analyses.

Soon thereafter, other research groups explored whether alternative ways to summarize molecular activity data in pathways could exploit the information content more effectively. Tomfohr *et al.* [28] showed that applying a dimension reduction approach, Singular Value Decomposition (SVD), to gene expression levels of pathway members and using the first eigenvector (called ‘meta-gene’) to derive a pathway activity representation, enabled the discovery of statistically significant pathway alterations in disease-related microarray data sets.

Because only a subset of pathway members may be involved in disease-associated changes, Lee *et al. [*29] later proposed to score pathway activity by focusing on condition-responsive genes (CORGs) within pathways. They applied a greedy search algorithm to find a gene subset that maximizes a *t*-statistic score for sample class discrimination, using a weighted sum of normalized gene expression levels for the selected input genes. On multiple microarray cancer data sets, the CORG-based pathway predictors provided increased discriminative power in comparison with gene-based classification, and the rankings of pathway alterations were more reproducible when tested across 100 alternative 2-fold splits of each data set.

A first probabilistic method to infer pathway activity from omics data was developed by *Su et al.* [30]. It estimates the log likelihood ratio (LLR) between different phenotype hypotheses (e.g. disease or control) from the expression level of each pathway member gene *i* (LLR_i_ = log[f_i_^1^(x^i^_j_)/ f_i_^2^(x^i^_j_)], where f_i_^y^(x) is the conditional probability density function of gene *i* under phenotype *y*), and then uses these ratios for a weighted combination of expression levels into a summarized pathway activity fingerprint a_j_ (a_j_ = Σ_i_^n^ LLR_i_ (x^i^_j_)), assuming independent contributions of the LLRs for different member genes. The authors compared their approach on two breast cancer data sets against gene-level predictors, the mean/median pathway summarization, the SVD- and CORG-based techniques described above, and their method achieved the highest predictive performance in both within-data set cross-validation experiments and one out of two cross-data set prediction experiments (the mean summarization scored higher in one case).

While the techniques discussed above mostly summarize pathway activity via (weighted) averages or dimension reduction approaches, it was noted that disease-associated pathway alterations may affect the variance of expression levels in pathways more strongly than the mean. Generally, variance patterns in disease-related omics data can reflect inter-patient variation (e.g. differences in the etiology or disease stage) or intra-patient fluctuations (e.g. on/off phases as those observed in Parkinson’s disease or imbalances in the activity of specific cellular pathways), and the study of both of these sources of variation may be important to improve the understanding of the disease. Glaab *et al.* [31] therefore proposed a variance-based approach to identify discriminative pathway signatures, providing a possibility to account for variation in the activity of cellular processes for a single patient and for variation across multiple individuals, and combined it with diverse classification approaches as part of a public Web application. Apart from modifying the pathway activity representation, they developed a rule-based learning method comparing activities in pairs of pathways rather than comparing activities of single pathways against fitted threshold values to increase the robustness of pathway-based classification models [37]. Rule-based models have high stability with regard to small variations in the input data and facilitate the biological interpretation of prediction models by using only combinations of simple if-then-else rules for sample classification [38].

A further strategy to improve classifier robustness is to use discretized pathway activity scores or counting statistics, e.g. Svenson *et al.* [32] presented an approach that first determines how many genes are up- or downregulated in a pathway using a predefined fold-change threshold. This information was combined into a ratio score, which equals 1 if all pathway members are upregulated, 0 if equal numbers of genes are up- and downregulated, and −1 if all genes are downregulated. After computing this ratio score for all pathway gene sets and training samples from a microarray case-control study, they applied nearest centroids classification to the pathway ratio scores to classify new test set samples. In comparison with gene-level classification, they report that the proposed classification approach performed better for all tested training set sizes.

### Graph-based and hierarchical pathway activity scoring

Cellular pathway diagrams do not only capture information on the functional relatedness of biomolecules involved in a pathway, but also on the network topology of regulatory or molecular interactions that link them together.

Efroni *et al.* [33] presented one of the first pathway analysis approaches, which not only scores pathway activities in omics data, but also the consistency of molecular activities within pathways, given the topology of interactions between their members. Considering the input and output genes for a regulatory interaction, their probabilities of being in an under- or overexpressed state in a condition of interest can be estimated from gene expression measurements, although noise in the data may make the estimates highly uncertain. A consistency score can then be obtained by comparing the state probabilities for output genes against their inferred state probabilities, derived from the estimated states of the interconnected input genes and the probability of the relevant interactions to be active or inactive. Thus, averaged pathway consistency scores can be computed and used as predictive features in combination with pathway activity scores to classify biological samples representing different conditions. Evaluating this approach on microarray cancer data by using the new pathway-level features as input for a Bayesian Linear Discriminant Classifier, the authors obtained similar or better predictive performance as compared with previously reported results for the test data.

Pathway topology information is also exploited in the approach by Vaske *et al.* [34], who converted cellular pathway diagrams into a factor graph model, describing the interactions between the involved biomolecules. The method can integrate information from different types of omics data for a biological sample and uses an Expectation Maximization (EM) algorithm to infer probabilistic pathway activity scores for each pathway/sample combination. When applying the approach to glioblastoma multiform gene expression and copy number data and grouping patients based on their pathway alterations, the identified subgroups displayed significant differences in quantitative survival in a Kaplan–Meier analysis. Robust patient stratification results were only obtained using the pathway-based scores and not via gene-level analyses.

Apart from considering the topology of molecular interactions within a pathway, studying alterations in all the downstream targets of pathway members (i.e. including targets not belonging to the pathway) may provide an additional source of information to further improve pathway activity scoring. Breslin *et al.* [35] presented a corresponding downstream analysis approach, which detects variation in pathway activity from the degree of correlation between downstream targets of a pathway. Using two correlations scores, one considering all pairs of downstream targets, and one considering only pairs without common transcription factors, they identified several differentially active pathways in microarray data from case-control studies. Additionally, they defined an activity score for pathways based on the average expression of their downstream target genes, and showed that the sample-wise activities for several pathways are significantly associated with clinical sample classifications.

Finally, although all approaches considered so far use pathway-level sample classification as a replacement for gene-level classification, these are not exclusive options. Kim *et al.* [12] proposed to build biomarker models from gene expression data at two levels, the gene and pathway level, via a hierarchical feature structure. This structure organizes the individual gene-level features into second-level aggregate features (pathways), used by a support vector machine (SVM) algorithm to classify samples. The authors considered three alternative approaches: GLEG (GSEA-based Leading Edge Gene feature method, using predictor genes derived from gene set enrichment analysis [19], which as a group maximally differentiate between the sample classes), GPF (GSEA Pathway Feature method, using enriched pathways as features) and SPF (SVM-based Pathway Feature method, using pathway features derived from SVM). When comparing these methods against classical gene-level and random gene set predictors, using cross-validation and cross-study prediction on microarray cancer data, in the majority of cases, the newly proposed methods achieved better results.

In summary, a wide range of pathway activity inference methods and machine learning approaches are available, which can be combined effectively for pathway-based diagnostic classification of biological samples. While pathway-based prediction does not necessarily provide superior predictive performance in within-data set cross-validation experiments, improved robustness and accuracy was observed consistently in cross-study prediction tasks.

## Network-based biomarker modeling

Although manually curated pathways have many benefits for the biological interpretation of large-scale omics data, in living cells, metabolic and signaling pathways are not isolated but interconnected within large and complex molecular and regulatory networks. These networks often include several genes, proteins or metabolites that are not annotated for any pathway and therefore ignored by pathway-based analysis methods. Consequently, to identify disease-associated modules of interconnected biomolecules in a more unbiased manner (i.e. without restricting the search space to biomolecules with known pathway annotations), network-based analysis methods have been introduced. While pathway-based approaches for biomarker modeling may have advantages in terms of model interpretability, the search space exploration in network-based biomarker discovery is not restricted by subjectively defined pathway boundaries, and the genome-scale molecular networks used as input typically cover significantly larger numbers of biomolecules than all combined pathways. Nevertheless, similar to subjectively defined pathways, networks assembled from public data sources suffer from various limitations, e.g. missing molecular interactions and lack of tissue-specific annotations, and these issues have to be addressed by dedicated methods (see section on ‘limitations and possible solution strategies’ below). In the following, two main types of network-based modeling approaches will be discussed: First, two-step sequential approaches, which score the activity in molecular subnetworks and afterward use these activities for predictive machine learning; and secondly, one-step network analysis approaches, which exploit network topology information directly within the predictive model building.

### Two-step network activity scoring and prediction approaches

Network activity over multiple interconnected biomolecules can be summarized and scored using similar averaging or dimension reduction approaches as in pathway activity scoring methods. However, in contrast to the straightforward use of predefined pathway definitions, first a molecular or regulatory network has to be assembled or reconstructed, using either public molecular interaction databases or applying network inference methods to omics data (in Table 2, an overview of different methodologies is shown, which are discussed in the following).

**Table 2.** Overview of network-based methods for machine learning analysis of omics data sequential network activity scoring and prediction methods are shown on top, whereas machine learning approaches using embedded network-based feature selection are listed below the bold black line

Methodology publication	Network activity/alteration scoring method	Prediction method
Tuck *et al.* [39]	Sample-specific gene regulatory networks are constructed and subnetwork activity is scored by summing over active interactions	Nearest neighbors, decision tree, Naïve Bayes, among others
Ma *et al.* [40]	Disease association is scored for genes based on gene expression data and their neighbors’ association scores in a PPI network using Markov Random Field theory	The approach is evaluated for disease gene prioritization but is applicable for predictive feature selection in combination with any prediction method
Chuang *et al.* [41]	Normalized gene expression data is mapped onto a protein interaction network and discriminative subnetworks are identified via a greedy search procedure	Logistic regression
Taylor *et al.* [42]	Hub nodes in protein interaction networks are determined and the relative gene expression of hubs with each of their interacting partners is computed to identify hubs with diverse relative expression across sample groups	Affinity propagation clustering is used to assign a probability of poor prognosis to breast cancer patients
Petrochilos *et al.* [43]	A random walk community detection algorithm is applied to discover modules in a molecular interaction network, and gene expression data is used to identify disease-associated modules	The approach is used to identify cancer-associated network modules and validated by scoring the enrichment of known cancer-related genes extracted from the OMIM database
Rapaport *et al.* [44]	Spectral decomposition of gene expression profiles is applied with respect to the eigenfunctions of a network graph, attenuating the high-frequency components of the expression profiles with respect to the graph topology	SVM

Li *et al.* [45]	A network-constrained regularization procedure for linear regression analysis is used to identify disease-related discriminative subnetworks	Penalized linear regression
Yang *et al.* [46]	Three machine learning methods for graph-guided feature selection and grouping are proposed, including a convex function and two non-convex formulations designed to reduce the estimation bias	Penalized least squares-based approach (GOSCAR: Graph octagonal shrinkage and clustering algorithm for regression)
Lorbert *et al.* [47, 48]	A sparse regression approach is proposed, using the PEN penalty to favor the grouping of strongly correlated features based on pairwise similarities (e.g. derived from a molecular interaction graph)	Penalized regression (PEN penalty)
Vlassis *et al.* [49]	Penalized logistic regression is applied using a convex PEN penalty function (see approach by Lorbert *et al.*) with absolute feature weights to better reflect the relevance of discriminative genes in the feature selection	Penalized logistic regression (PEN penalty with absolute feature weights)

A first method to construct new sample-specific gene regulatory networks for transcriptomics sample classification was proposed by Tuck *et al.* [39]. The networks were generated by determining the graph-theoretic intersection between a static connectivity network (representing transcription factor binding to gene promoter regions), obtained using data from the TRANSFAC database [50], with sample-specific coexpression networks (representing transcription-factor–target gene coexpression), derived from gene expression data. To extract discriminative features for diagnostic specimen classification from these networks, they proposed a link-based classification approach, comparing the activity status of gene regulatory interactions (called ‘links’) across different sample groups, and a degree-based classification method, comparing topological centrality measures [51] for the networks. When testing these approaches on data from different cancer case-control studies, high cross-validated accuracies were reported for both cell type and patient sample classification. Moreover, the network-based analysis enabled the authors to identify key transcriptional regulators altered under specific disease conditions.

Instead of constructing new regulatory networks, discriminative disease-associated network alterations can also be identified by computationally mapping omics data onto *in* *silico* representations of biochemical protein–protein interaction (PPI) networks. Ma *et al. [*40] developed a corresponding approach to obtain more reliable disease association scores for genes by exploiting neighborhood information from a PPI network. They used a modified Pearson correlation coefficient to assess the association between microarray gene expression and numeric values encoding the disease status of the samples (taking into consideration that these phenotype values may not have a normal distribution) and assigned the Fisher-transformed gene-phenotype association scores to the corresponding proteins in a PPI. Next, they recalibrate these association scores by modeling the underlying true scores for each gene using Markov Random Field theory [52], reestimating their values from weighted contributions of their network neighbors’ original association scores (the weights are determined according to different network neighborhood definitions, using either direct neighbors, shortest path or diffusion kernel neighborhoods, see [40] for details). When evaluating the utility of the recalibrated scores for disease gene prioritizations on microarray data using known Gene Ontology functional annotations, conventional prioritization approaches using only gene expression or PPI data were outperformed (although the scoring approach could also be used for predictive model building, this particular application was not considered).

While the approach by Ma *et al.* focuses on improving the disease-association scores for individual genes, Chuang *et al.* [41] presented a method identifying and scoring entire disease-related subnetworks, similar to their pathway association scoring approach discussed above (see *Lee et al.* [29]). After computing the mutual information (MI) between sample phenotype values (encoding the presence or absence of a disease) and discretized expression values for each gene from a microarray data set assigned to the proteins in a PPI, they applied a greedy search to expand the seed nodes in the network with locally maximal MI scores. Specifically, each seed node was expanded such that the sum of scores for the expanded network module is maximized (the search stops when no extension increases the total score above a predefined improvement rate). When training logistic regression classifiers on the normalized and averaged activities of the resulting subnetworks for breast cancer data, the authors found that the subnetwork markers were more reproducible than single-gene markers and provided higher accuracy in distinguishing metastatic from nonmetastatic tumors.

As an intermediate solution between focusing on individual biomolecules and entire network modules, Taylor *et al.* [42] proposed a method that investigates network nodes with outstanding topological properties and their direct neighbors. After computationally mapping breast cancer gene expression data onto the *in* *silico* representation of a PPI network, they determined proteins with large numbers of biochemical interaction partners (so-called ‘hub nodes’), and computed their relative expression compared with each of these interacting partners. They then determined for which hubs the relative expression differed significantly between long-term survivors and patients who died from the disease, and applied a clustering approach to assign a probability of poor prognosis to new patient samples (the specific method used is known as ‘affinity propagation clustering’ in the literature). The approach was evaluated using 5-fold cross-validation, providing accuracy, sensitivity and specificity estimates that compared favorably with the reported results for commercially available genomic breast cancer diagnostics.

Instead of considering topological properties of individual nodes in a molecular network, information from a network graph can also be extracted via algorithms for finding sub-graphs, which stand out in terms of their high density of molecular interactions (using approaches referred to as ‘community identification’ or ‘graph clustering’ methods in the literature). Petrochilos *et al.* [43] proposed a corresponding approach, which first applies a graph-based random walk algorithm on a genome-scale molecular network. Information from cancer-related gene expression data was then integrated into the network by setting the weight of each network node as the maximum fold change of probes corresponding to its gene symbol (weights for biochemical interactions are determined by the square of the mean of the absolute fold changes of the relevant interaction partners). Finally, the score of a network module of connected nodes was obtained by comparing its cumulative activity (i.e. the square of the average weighted expression for all its nodes) against a bootstrap distribution of cumulative activities obtained via random sampling of a matched number of fold change values. When testing the enrichment of known cancer genes in the top-scored network modules identified with this approach, a similar or better performance was reached in comparison with other widely used module-finding algorithms (potential alternative applications of the identified modules for biomarker modeling were not assessed in this publication).

Apart from averaging molecular activities over network neighborhoods or using community identification methods, signal processing techniques may provide a further means to glean useful information from a network for predictive model building, as shown in an approach by Rapaport *et al.* [44]. They made use of the observation that genes in close proximity to each other in a network tend to have similar expression and proposed to denoise microarray measurements by removing their high-frequency component over the network. For this purpose, spectral decomposition of gene expression profiles with respect to a molecular network graph was applied, followed by attenuating high-frequency signal components, expected to represent measurement noise. The method was evaluated for supervised analysis of irradiated and nonirradiated yeast strains using a SVM, providing similar classification performance as a model built without the network-based filtering, but facilitating biological data interpretation by grouping the selected biomolecules according to their participation in the network modules.

### One-step machine learning approaches for network analysis

In contrast to the network analysis approaches considered so far, which apply network feature extraction and predictive machine learning analysis in separate steps, more recently, one-step network-based feature selection approaches have been proposed, integrating the attribute selection directly into the predictive model building. Most of these approaches formulate the model building task as an optimization problem formulation, in which the objective function for classification or regression is extended by a penalty term promoting the selection of grouped features in a molecular network (this strategy is also referred to as network-constraint regularization).

Li *et al.* [45] proposed one of the first corresponding approaches by adding a penalty term to linear regression, incorporating network information into the analysis via the Laplacian matrix of the network graph. The approach penalizes the L1-norm of the feature weights and encourages a smooth profile of weights over neighboring nodes in the network. However, Binder and Schumacher later reported that the method has lower performance than a null model, i.e. a model not using any covariate information [53]. As possible explanations, they note that Li *et al.* discarded censored observations and about 20 000 variables that could not be assigned to corresponding nodes in the molecular interaction network (see section on ‘limitations’ below). Yang *et al. [*46] suggested that the previously used network grouping penalties can introduce additional estimation bias into the model when the coefficient signs for two features connected in the graph are different. They presented alternative penalties to achieve network grouping and sparse feature selection, in particular two non-convex penalties, which shrink only small differences in absolute values of feature weights to reduce the estimation bias [46]. In experiments on synthetic data and two real data sets, the new approaches outperformed previous feature grouping methods.

However, with non-convex penalties, finding global optimal solutions is often not feasible and even identifying good local optima may require high computational effort. Lorbert *et al.* [47, 48] proposed an alternative generic convex penalty, the Pairwise Elastic Net (PEN), that provides sparse feature selection and promotes the grouping of attributes according to a user-defined feature similarity measure (e.g. obtained from biochemical interaction weights in a molecular network). PEN is a generalization of the Elastic Net, a method providing a trade-off between L1- and L2-penalized regressions by an adjustable parameter. In PEN, this parameter can be replaced to determine the trade-off using additional information from an attribute similarity matrix (different instances of PEN can be defined as long as the similarity matrix is positive semidefinite and non-negative). Comparing PEN against other popular machine learning approaches on simulated data with a grouping structure among the features, PEN achieved a competitive mean squared error (MSE) and provided sparser solutions than approaches with similar MSE.

More recently, Vlassis *et al.* introduced a new instance of PEN, which penalizes differences between the ‘absolute’ values of weights of interlinked features in a network graph. The motivation behind this approach, termed GenePEN, is that the magnitude of a weight in a linear model reflects the predictive value of the corresponding variable, so that the weights for irrelevant features are driven to zero by the penalty. By ensuring the convexity of the penalty function, global optimal solutions can be identified efficiently with existing optimization frameworks. When evaluating GenePEN on simulated data and real-word microarray data sets, in comparison with other classification methods using feature grouping, the method provided similar predictive power and gene selections, sharing significantly more connections within a molecular interaction network. Visualization of the corresponding subnetworks enabled a biological interpretation of disease-affected network regions, which were enriched in known disease-related genes obtained from literature mining.

Overall, network-based sample classification methods provide a new means to analyze complex omics data sets, allowing researchers to identify coherent molecular network alterations under different biological conditions. Identifying such network-level patterns in omics data for diseases with complex molecular manifestations can shed new light on the molecular mechanisms of the disease and facilitate the development of robust multifactorial biomarker signatures.

In contrast to single-molecule-based biomarker modeling, a network-level approach has the potential to capture diverse facets of a heterogenic disease reflected via alteration patterns in different network regions. In comparison with pathway-based machine learning approaches, methods using genome-scaled networks as prior knowledge may produce models that are more difficult to interpret biologically, but which can identify a much wider range of alterations in cellular processes (covering many genes, proteins or metabolites without any known pathway annotations). Finally, network- and pathway-based classification approaches share the main benefit of improving model robustness in cross-study analyses as compared with using individual biomolecules as features. Among these new higher-level biomarker signatures, network-based signatures accounting for molecular activities over larger and algorithm-derived network regions may often provide more robust multifactorial markers than signatures for smaller pathways, which are typically defined subjectively, possibly overlooking relevant functionally related molecules in the surrounding network. However, the model robustness will also depend on other factors, e.g. the occurrence of protein complexes in the studied pathway/network (members of these complexes tend to have highly coordinated activity, providing more robust averages) and the reliability and completeness of the specific network or pathway data source used (see limitations discussed in the following section).

## Limitations and possible solution strategies

While pathway- and network-based analyses of omics data can enrich the biological interpretation of complex molecular alteration patterns and facilitate robust biomarker modeling, users should also be aware of common limitations behind these methods and possibilities to address them.

Although detailed functional annotations have been collected for a large portion of genes in humans and common model organisms, many identified genes still lack any functional assignment. Consequently, a significant portion of genes and proteins analyzed via current high-throughput measurement techniques can often not be assigned to any pathway or molecular network for systems-level analyses. Similarly, several gene regulatory interactions and PPIs are still unknown, and experimental techniques to identify molecular interactions may also generate false-positive results [54]. Thus, public molecular interaction databases only cover a subset of biochemical interactions occurring in living cells, and the reliability of reported interactions varies depending on the amount and type of associated evidence.

A further limitation of current pathway databases and assembled molecular networks is that tissue- and cell-type specificity of molecular interactions and protein functions is typically not considered in these data resources. This can be particularly problematic when studying diseases showing selective vulnerability for specific tissues or cell types, e.g. as found in many neurodegenerative disorders [55]. Similarly, in many biomarker profiling studies and public omics data sources, concomitant data from different tissue and cell types for a disease of interest is lacking, as well as longitudinal data required to identify relevant dynamic changes during disease pathogenesis and progression. Moreover, a variety of limitations related to the specific origin, processing and storage of specimens collected for biomarker studies may negatively impact subsequent analyses and the comparability of data from different studies. For example, studies might differ in terms of the sample storage durations, in terms of whether the reference tissue for tumor tissue is derived from the same patient or from an unaffected control individual, whether a preinfarction specimen is accepted as a reference for the specimen obtained on infarct suspicion, or whether laser capture microdissection is applied to isolate specific cells of interest rather than using heterogeneous cell populations.

To account for the varying reliability of public biochemical interaction data when assembling a molecular network, various approaches to determine confidence scores have been proposed [56–59]. One of the most comprehensive resources is the STRING PPI database, which allows users to filter and extract biochemical interactions for a wide range of organisms using different sources of evidence, as well as a combined confidence score [60, 61].

A frequently encountered difficulty in pathway analyses is also the conversion of the information content of public pathway diagrams into adequate data structures for computational investigations. Pathway structures usually contain more complex layers of information than gene–gene networks, e.g. meta-nodes representing protein complexes or gene families, and catalysis reactions represented by edges pointing to edges. Dedicated computational approaches are therefore required to convert pathway diagrams into uniform network representations, e.g. using biology-driven rules as in the R/Bioconductor software package ‘graphite’ [62].

Apart from this conversion task, building tissue-specific pathway/network representations or assigning probabilities for tissue specificity to the biological interactions in an existing network is a further challenging problem because interactions are rarely confirmed experimentally in multiple specific tissues. Most bioinformatics approaches for assigning tissue specificity scores to protein interactions use publicly available gene or protein expression data from large-scale tissue profiling studies [63, 64], e.g. predicting the potential for a molecular interaction to occur in a certain tissue depending on whether both interaction partners are expressed in this tissue [65]. Moreover, dedicated pathway- and network-analysis methods have been developed for tissue-specific disease gene prioritization and gene set enrichment analysis [23, 66], which may also provide useful filters for feature selection in machine learning methods for sample classification.

While changes in the mean or variance of the overall molecular activity in pathways can provide robust biomarkers for some diseases, pathways may also contain forks, and only one of their branches or stems may be affected in a pathological condition. Approaches focusing on changes in the overall pathway activity may therefore fail in detecting significant alterations affecting only one pathway branch. Although network analysis methods identifying altered subnetworks may be helpful in this situation (using a pathway graph as input instead of a genome-scale molecular network), ideally, available knowledge on distinct pathway branches or outstanding regions in a complex pathway architecture should be exploited directly, e.g. by partitioning a pathway into corresponding ‘subpathways’ and separately analyzing their molecular alterations using existing pathway-level bioinformatics tools.

Another challenging and still largely unsolved problem in the analysis of alterations for pathways, networks and single biomolecules is the distinction between omics changes representing causes or effects (or ‘drivers’ or ‘passengers’) of a disease. Apart from philosophical disagreements on how causation should be defined [67], the discrimination between correlated events and causally linked events on high-throughput omics data sets is typically severely hampered by large numbers of potential confounding variables and complex nonlinear relationships between the attributes. Thus, conclusive evidence for causal links can usually only be obtained from *in* *vitro* and *in* *vivo* perturbation experiments rather than from high-throughput data analysis, even when using high-quality time-series data with large sample sizes and dedicated statistics for identifying cause–effect relationships (e.g. the Granger causality test [68]). Prior biological knowledge, e.g. on transcription factor–target relationships, can however enable the construction of causal graphs to check whether omics measurements are consistent with certain causal molecular hypotheses or to prioritize corresponding hypotheses for experimental validation [69]. Bill Shipley has written a dedicated book on methods for causal analysis of observational data in biology [67]. These approaches are not only relevant for identifying drug targets and investigating disease etiology and progression, but may also provide useful information for the biological interpretation of diagnostic biomarker models.

Improving incomplete pathways and networks by identifying missing molecular interactions is arguably one of the most challenging problems in this domain. Pathway definitions are typically created in a subjective manner because no universal predefined criteria for determining pathway membership and boundaries are available, and the curators of the corresponding databases are required to make judgments on the relevance of potential pathway members. These subjective definitions may be prejudiced and erroneous, and even networks assembled automatically from public databases may contain biases, e.g. resulting from prior subjective decisions on which putative PPIs are of sufficient interest for experimental validation. Moreover, in diseases the pathway topology may change, and generic pathway databases have recently been complemented by dedicated pathway maps for specific human disorders, e.g. AlzPathway [70] for Alzheimer’s disease and PDMap [71] for Parkinson’s disease. Because these disease-related pathway maps typically cover the biomolecules involved in a specific disorder and the potentially altered network topology more comprehensively and accurately than generic pathway maps, disease-specific pathways should be considered as the preferred resource for pathway-based diagnostic biomarker modeling (in the specific case of a known disease-associated topology alteration, ideally, a corresponding pathway map with unaltered topology should be used additionally as a reference in the modeling process).

Moreover, dedicated bioinformatics techniques have been developed to identify ‘network rewiring’ events, e.g. broken chains or new interactions changing the pathway/network topology, which remain undetected by conventional pathway analyses. Instead of assuming that molecules close to each other in a network undergo similar changes in a disease, these approaches identify alterations in the correlations between the activities of these molecules as signatures for potential pathological changes in network topology [72–74]. However, the top-scoring results require experimental verification, e.g. using *in* *vitro* disease models, and the success may strongly depend on whether reliable prior pathway knowledge is available.

To support relevant manual pathway curation efforts, computational approaches for *ab initio* discovery of pathways within genetic or molecular networks have been proposed [75–78], as well as bioinformatics methods to extend existing pathway definitions according to objective criteria for pathway compactness and connectedness [79]. Moreover, various machine learning approaches for predicting unknown PPIs using public structural and functional information have been developed [80–84], e.g. the authors of the PrePPI method report validation results suggesting their prediction algorithm is comparable in accuracy to high-throughput experiments [85]. For proteins with unknown structure and few functional annotations, these *in silico* prediction approaches are however still bound to provide limited reliability, and only high-confidence biochemical interactions should be included in networks for omics data analysis.

Finally, to reduce the influence of false-positive predictions on network analyses, molecular interactions should not only be prefiltered by confidence when assembling the network, but also weighted by confidence to apply analysis techniques exploiting these weights to score the reliability of identified network alterations. In subsequent biological validation experiments, investigators can then focus on verifying only the most reliable network alteration patterns derived from the *in silico* analyses.

## Translating multifactorial marker models into diagnostic tests

### Pathways from omics research discoveries to clinical diagnostic assays

High-throughput omics measurement techniques are typically not designed for diagnostic applications, but for broad systems-level analyses, hypothesis generation and the construction of first tentative machine learning models for sample classification. Such tentative models require subsequent refinement and validation using more sensitive and reproducible measurement techniques to evaluate their potential for diagnostic applications. For example, a sample classification model built and cross-validated using microarray gene expression data, with an embedded feature selection to choose only the most informative genes as predictors, can be validated using more accurate quantitative reverse transcription polymerase chain reaction (qRT-PCR) measurements for the subset of chosen genes.

Importantly, to avoid wrong conclusions in the evaluation of diagnostic classification models, adequate statistical methods have to be chosen to assess a model’s overall predictive performance (quantifying how close predictions are to the actual outcome), its calibration/reliability (measuring how close to x of 100 individuals with a risk prediction of x% have the outcome) and its discriminative ability (determining whether individuals with the outcome have higher risk predictions than those without) [86]. Owing to the inherent uncertainty associated with diagnostic tasks, predictions should be provided in a probabilistic, rather than deterministic, form [87], and the overall performance should be quantified using so-called ‘proper scoring rules’, for which the expected score is optimized when the predictive distribution agrees with the true distribution of the quantity to be estimated (a corresponding example is the Brier score for binary and survival outcomes [88]). Conversely, the optimization of models with respect to conventional discontinuous non-error rates like the percentage correct classification can provide misleading results, e.g. when the predicted probabilities are close to the chosen decision threshold required for these measures [89]. To assess the calibration of a model, the Hosmer–Lemeshow ‘goodness-of-fit’ test can be used [90], and the concordance statistic to quantify the discrimination ability [91]. If a reference prediction system is already available, dedicated measures of the relative improvement achieved with a new prediction method should additionally be computed (referred to as the ‘skill’, e.g. quantified via the Brier Skill Score [92]). Moreover, decision-analytic approaches like decision curve analysis [93], designed to assess the net benefit achieved making decisions according to model predictions, should be applied if the model is to be used to direct clinical patient management [86].

For the study design, initial power calculations are required to ensure that sufficient sample sizes are available for all statistical assessments [94]. This also involves choosing an adequate splitting of the measured data into training, test and validation sets and selecting suitable cross-validation or resampling techniques for model optimization and evaluation (e.g. using two-level external cross-validation [95]) [96].

Importantly, clinical validation does not only necessitate significantly larger sample sizes than most research studies, but also independent replication tests on data from other patient cohorts, the clear specification of the biological rationale behind the method and a demonstration of its clinical utility. In contrast to the regulatory framework for drugs, there are multiple pathways for the translation of omics-based tests into validated *in vitro* diagnostic test devices. These tests can be developed and validated either through review by the Food and Drug Administration (FDA) or through validation and performance by a specific laboratory certified according to Clinical Laboratory Improvement Amendments (CLIA) [97].

**Figure 1.**
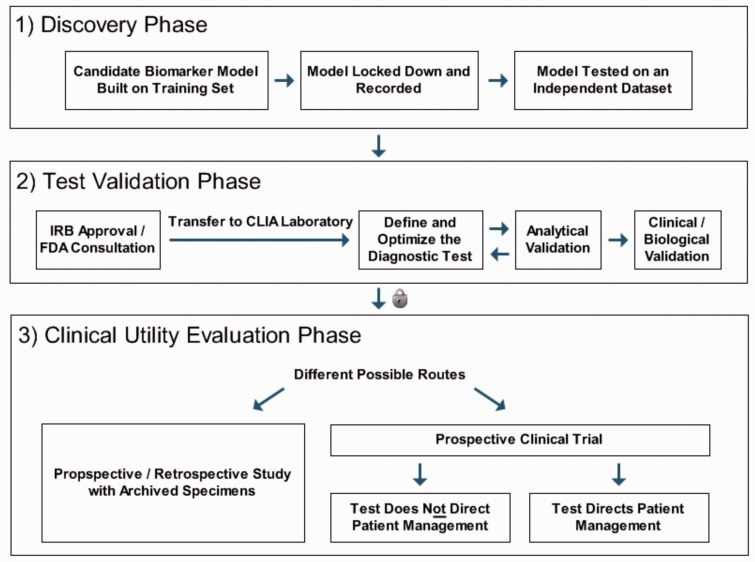
Example illustration of common stages during the development of omics-based diagnostic tests (simplified version of the process presented in a study by the US Institute of Medicine [97], focusing on the major steps in the pipeline). After the transition from the second to the third phase (highlighted by the lock symbol), the diagnostic test must be fully defined, validated and locked down. Many important variants and alternatives to the outlined example process exist, as well as different realizations of generic steps in the process (e.g. cases in which a test directs patient management may cover different situations, depending on whether clinicians are free to use the test result as they see fit, or whether predefined procedures have to be followed subject to contraindications and/or subject to the test results). The setup may also vary depending on whether it is known exactly how patients would have been treated had they been randomized to the opposite arm, depending on whether the test entails a treatment delay, and whether the adequate cutoff threshold for the test is uncertain.

Because using established medical product development pipelines as in pharmaceutical companies is not common practice in academia, for many biomedical research institutions an early collaboration with an experienced industrial partner is often advisable. Although currently no unique and widely recognized standard process for translating omics research findings into clinical diagnostics is available, common recommendations by widely recognized health organizations can be followed. In particular, a committee by the US Institute of Medicine has conducted a study on omics-based clinical test development and proposed a generic process for the development and evaluation of these tests as a recommended guideline [97]. A corresponding example process, which is briefly outlined for illustration purposes in Figure 1 and not meant to cover all important variations, starts with the discovery phase in which a candidate biomarker model is built on a training set, locked down and evaluated on a test data set (this set of samples should be completely independent from the training set). In the following test validation phase, after institutional review board approval and consultation with the FDA, a CLIA-certified laboratory defines and optimizes the diagnostic test method, clinically and biologically validates the test on a blinded sample set and implements the test according to current clinical laboratory standards.

Interestingly, the authors of the guideline highlight that a frequent shortcoming of omics-based tests is the lack of a biological rationale behind the test—while single-molecule markers are often known to play a role in the disease, multifactorial omics models obtained from machine learning are often more difficult to interpret and involve a greater risk of overfitting. New pathway- and network-based modeling techniques as discussed in this review could therefore help to address some of these shortcomings and provide more interpretable and robust models as opposed to classical ‘black box’ machine learning models.

In the following stage of the clinical development process, the locked-down test is evaluated for clinical utility via one of the following approaches: (i) A prospective–retrospective study using archived specimens from previous clinical trials, (ii) a prospective clinical trial in which the test (a) directs patient management, or (b) does not direct patient management [97]. The complexity and duration of a corresponding clinical study or trial will largely depend on the specific type of biomarker developed and the proposed clinical benefit. For diagnostic biomarkers focused on in this review, procedures may vary significantly depending on whether the test is designed to detect the presence, severity or the subtype of a disease. Prognostic biomarkers, which indicate the future clinical course of a patient with regard to a specific outcome, and predictive biomarkers, which predict the responders and the extent of susceptibility to a particular drug effect, will also require different development and evaluation procedures than diagnostic markers. Finally, for each type of biomarker, different clinical benefits may be envisaged and significantly influence the design of a study, e.g. the goal to choose more appropriate treatment options, or the objective to diagnose the disease earlier to enable more effective therapies to prevent, halt or slow down its progression.

### Previous success stories in omics-based development of diagnostic assays

A variety of multifactorial, omics-based biomarker models have been translated successfully into diagnostic tests in recent years, in particular, in the field of cancer sub-type stratification. A prominent example is the Oncotype DX test to assess a woman’s risk of recurrence of early-stage, estrogen-receptor-positive breast cancer and the likelihood of benefitting from chemotherapy after surgery. This test measures the activity of 21 genes in tumor samples and then determines a recurrence score number between 0 and 100 (higher scores reflect greater risk of recurrence within 10 years). As opposed to other diagnostic tests using frozen samples, the Oncotype DX assay uses tumor tissue samples that are chemically preserved and sealed in paraffin wax (see [98, 99] for details on the sample collection and analytics).

The development of Oncotype DX involved typical steps of an omics biomarker profiling and top-down filtering approach: first, by analyzing the entire transcriptome on high-throughput microarray data and using knowledge from the literature and genomic databases, 250 candidate marker genes were selected [98]. The relation between the expression of these candidates and the recurrence of breast cancer was then assessed in data from three independent clinical studies on 447 patients. The results were used for a final filtering, providing a panel of 16 cancer-related genes and 5 reference genes, whose expression levels enabled the calculation of recurrence scores for tumor samples via a machine learning model. This diagnostic approach was successfully validated in multiple clinical studies and has been included in the treatment guidelines for breast cancer by the National Comprehensive Cancer Center Network and the American Society of Clinical Oncology.

While in the case of the Oncotype DX test, the set of required markers could be narrowed down to a small number of genes with prior knowledge on their relationship to the disease, for other complex and more heterogeneous diseases, significantly larger numbers of molecular predictors may be needed for accurate diagnosis. In such cases, pathway- and network-based modeling approaches may facilitate the generation of robust and biologically interpretable models, which could afterward undergo similar diagnostic test development and validation procedures as the initial model behind the Oncotype DX assay. Importantly, the success of the Oncotype DX approach is not an isolated case, but other commercial diagnostic assays have been developed and validated using similar strategies, including MammaPrint [100], Prosigna (PAM50) [101], Mammostrat [102], Tissue of Origin [103], AlloMap [104], Corus CAD [105] and OVA1 [106], among others.

In summary, successful translation of omics-based biomarker models into clinically accepted commercial diagnostic tests has been achieved in multiple cases in the past. Given a large number of complex diseases for which more reliable, earlier and cheaper diagnostic tests are still needed, there is a significant potential to develop improved approaches using omics-based biomarker modeling and exploiting prior biological knowledge from pathways and molecular networks.

## Conclusions

For diseases with complex molecular manifestations, omics-based biomarker models have the potential to better reflect clinically relevant aspects of their heterogeneity than classical single-molecule diagnostic markers. However, various statistical limitations in high-dimensional data analysis, often resulting in a high risk of overfitting and restricted biological interpretability of standard ‘black box’ machine learning models, can hamper the progress in developing new clinically useful biomarker signatures.

Among possible strategies to reduce model complexity and integrate prior biological knowledge into the model building procedure, cellular pathway- and molecular network-based prediction methods provide promising new routes toward more reliable omics-based biomarker signatures. A wide variety of bioinformatics approaches have already been developed in recent years to identify disease-associated pathway and network alterations in omics data and use them to classify new samples. While public molecular networks and pathway definitions also have specific limitations that need to be addressed by the user, e.g. by assigning confidence weights to molecular interactions, pathway- and network-level predictive analyses often lead to classification models with improved interpretability and robustness in cross-study prediction tasks. These new systems-level approaches will therefore provide a valuable addition to the existing toolset for omics-based biomarker modeling.

Key Points

- Omics-based machine learning models for diagnostic specimen classification often lack biological interpretability and have limited cross-study robustness and accuracy. Integrating prior knowledge from cellular pathway and molecular network into these models can help to address these shortcomings.
- Public pathway and molecular interaction databases cover only a subset of the biochemical interactions occurring in living cells, typically lack annotations on tissue-specific reactions and may also contain errors. When applying pathway or network analysis methods, the user should be aware of these limitations and the different options to address or alleviate them, e.g. by using confidence weighted interactions and estimating their tissue specificity using public gene or protein expression data.
- Pathway- and network-based machine learning methods for diagnostic specimen classification are characterized by different strengths and weaknesses. While pathway-based approaches have the benefit of providing models with high biological interpretability that take advantage of human expert knowledge on disease-relevant cellular processes, their coverage is limited to biomolecules with known annotations. On the contrary, network-based models are typically more difficult to interpret, but can exploit information on a much wider range of biomolecules.

## Funding

This work was supported by the Fonds Nationale de la Recherche, Luxembourg (grant no: C13/BM/5782168).
